# TRIzol treatment of secretory phase endometrium allows combined proteomic and mRNA microarray analysis of the same sample in women with and without endometriosis

**DOI:** 10.1186/1477-7827-8-123

**Published:** 2010-10-21

**Authors:** Amelie Fassbender, Peter Simsa, Cleophas M Kyama, Etienne Waelkens, Attila Mihalyi, Christel Meuleman, Olivier Gevaert, Raf Van de Plas, Bart de Moor, Thomas M D'Hooghe

**Affiliations:** 1 Leuven University Fertility Centre, Department of Obstetrics & Gynaecology, University Hospital Gasthuisberg, Leuven, Belgium; 2 Division of Reproductive Health and Biology, Institute of Primate Research, P.O. Box 24481-00502 Karen, Nairobi, Kenya; 3 Biochemistry Section, Department of Molecular Cell Biology, Campus Gasthuisberg, Leuven, Belgium; 4 Department of Electrical Engineering, ESAT-SCD, K.U.Leuven, Kasteelpark-Arenberg 10, B-3001 Heverlee, Belgium

## Abstract

**Background:**

According to mRNA microarray, proteomics and other studies, biological abnormalities of eutopic endometrium (EM) are involved in the pathogenesis of endometriosis, but the relationship between mRNA and protein expression in EM is not clear. We tested for the first time the hypothesis that EM TRIzol extraction allows proteomic Surface Enhanced Laser Desorption/Ionisation Time-of-Flight Mass Spectrometry (SELDI-TOF MS) analysis and that these proteomic data can be related to mRNA (microarray) data obtained from the same EM sample from women with and without endometriosis.

**Methods:**

Proteomic analysis was performed using SELDI-TOF-MS of TRIzol-extracted EM obtained during secretory phase from patients without endometriosis (n = 6), patients with minimal-mild (n = 5) and with moderate-severe endometriosis (n = 5), classified according to the system of the American Society of Reproductive Medicine. Proteomic data were compared to mRNA microarray data obtained from the same EM samples.

**Results:**

In our SELDI-TOF MS study 32 peaks were differentially expressed in endometrium of all women with endometriosis (stages I-IV) compared with all controls during the secretory phase. Comparison of proteomic results with those from microarray revealed no corresponding genes/proteins.

**Conclusion:**

TRIzol treatment of secretory phase EM allows combined proteomic and mRNA microarray analysis of the same sample, but comparison between proteomic and microarray data was not evident, probably due to post-translational modifications.

## Background

Endometriosis is a gynaecological disorder, defined as the presence of endometrial-like tissue outside the uterus and is associated with chronic intrapelvic inflammation. Its symptoms can impact on general well-being [1] and include severe dysmenorrhoea; deep dyspareunia; chronic pelvic pain; cyclical or premenstrual symptoms (e.g. bowel or bladder associated) with or without abnormal bleeding; infertility and chronic fatigue.

Well established biological differences between eutopic endometrium from women with and without endometriosis represent an interesting scientific basis to develop a semi-invasive diagnostic test for endometriosis based on these differences. Recent evidence suggests that significant biological differences between eutopic endometrium from women with and without endometriosis [2] may offer the basis for a semi-invasive diagnostic test based on the analysis of an endometrial biopsy. Numerous proteomic [3-10] and mRNA microarray [11-14] studies have demonstrated important biological differences between eutopic endometrium from women with and without endometriosis. Furthermore, data from other investigators [15-17] and from our group [18] suggest that endometriosis can be diagnosed based on the increased endometrial density of nerve fibres in women with endometriosis compared to controls.

The primary aim of this study was to test the hypothesis that TRIzol extraction of endometrium enables a combined mRNA microarray and proteomic analysis of the same EM sample from both women with and without endometriosis. The secondary aim of our study was to compare proteomic data (presented in this study) with mRNA microarray data [13] of the same EM sample.

## Methods

### Patient selection

The same endometrium samples selected for this study were as those used in our previous microarray study [13]. Briefly, the biobank of the Leuven University Fertility Centre was searched to identify 16 endometrial (EM) samples obtained during the secretory phase (dated between day 23 - 26) from each of the following 3 groups, women with a normal pelvis (controls, n = 6), women with minimal to mild endometriosis (stages I-II, n *= *5), and from women with moderate to severe endometriosis (stages III-IV, n= 5). Endometriosis was staged according to the classification system of the American Society of Reproductive Medicine [19]. Endometrial samples had been collected during hysteroscopy/laparoscopy procedures for either infertility or pain and had been frozen at -80°C until use. None of our patients took oral contraceptives or other hormonal treatment for endometriosis within 3 months prior to EM sample collection. Women with and without endometriosis had the following age (mean 28.10±2,767, median 27.5, range 26-29.5 years) and (mean 32.33±3.933, median 33, range 28.50-35.5 years), respectively. All patients had signed a written informed consent before surgery and had agreed on the collection of tissues for research. The study protocol had been approved by the institutional ethical and review board of the University Hospital Gasthuisberg for the protection of human subjects.

### Preparation of endometrial samples

TRIzol (Invitrogen Life Technologies, Carlsbad CA, USA) [20], a monophasic solution of phenol and guanidine isothiocyanate, was used as a one step reagent for the extraction of RNA, Protein from each EM sample. The protein quantity and quality was analyzed on the Nanodrop and protein measurements ranged between 0,39 to 16 mg/ml. 10 μg of protein concentration were used to spot on each surface.

Frozen EM tissue biopsies were immediately thawed in TRIzol reagent according to the instructions of the manufacturers. Briefly, tissues were homogenized using a glass tube with a glass stick to smash the sample to pieces, 300 μl/105 μl chloroform were added and left at room temperature for five minutes. The samples were centrifuged at 10 000 rpm at 4°C for 15 minutes. The upper aqueous phase was separated and washed with Qiagen kit (following manufacturer's instructions). The aliquot including the protein fraction were thawed on ice and sonicated. 600 μl acetone was added with inversion and left at room temperature for 10 min. The samples were centrifuged at 12000 rpm at 4°C for 10 minutes. Twice 0.5 ml of 0.3 M urea in 95% ethanol was added and left at room temperature for 10 min. The samples were centrifuged at 8,000 g for 5 minutes and twice 1 ml of urea/ethanol were added and dissolved in 300 μl U9 Ciphergen (9 M Urea, 2% 3-[(3-cholamidopropyl) dimethyl-ammonio]-1-propanesulfonate (CHAPS), 50 mM tris(hydroxymethyl)aminomethane-HCl (Tris-HCl) pH 9.0) (Ciphergen Biosystems, Fremont, CA, USA). The samples were sonicated and left at room temperature for 10-20 minutes and spun at 8,000 g for 5 minutes to sediment the insoluble protein. The samples were stored at -80°C.

### ProteinChip Arrays

First a brief test phase was performed on four chip types (Cm10, IMAC30-Cu, H50 and Q10) to find the best (rich spectra) chip type for this experiment. Two different chip surfaces with distinct chromatographic properties and binding affinities were used, Weak cation exchange surface (CM10) with a low stringency binding buffer (50 mM NaOAC, pH 4.0) and Immobilized metallic affinity capture surface (IMAC-30-Cu) loaded with CuSO_4_, with a 0.1 M phosphate, 0.5 M NaCl, pH7.0.

Briefly, ProteinChip array spots were equilibrated with 150 μl of respective binding buffer (Ciphergen, Fremont, CA, USA) while shaking twice for five minutes at room temperature to pre-activate binding surfaces. Then, 20 μl of sample (10 μg per spot) diluted (1:5 vol/vol) with the surface-type dependent binding buffer were loaded onto each spot in duplicate and incubated for 60 minutes at room temperature while being shaken in the dark (MicroMix5, form 20, amplitude 5; Diagnostics Product Corporation, Gwynedd, Wales, United Kingdom). The unbound proteins/peptides on the ProteinChip array surfaces were washed away with appropriate buffer twice for 5 minutes rinsed in 150 μl of Milli-Q water and air-dried. Mass spectra of the retained proteins were obtained by ionising the proteins using two types of energy absorbing molecules (EAM): alpha-cyano-4-hydroxy cinnamic acid (CHCA), for small molecules (< 15 kDa), and sinapinic acid (SPA), for larger molecules (both EAM were obtained from Ciphergen, Fremont, CA, USA). The CHCA (5 mg CHCA dissolved in 150 μl of 50% acetonitrile, 0.5% trifluoroacetic acid) was diluted five times in the respective solvent, and 1 μl was applied twice onto the retained proteins on the spots. The SPA (5 mg SPA dissolved in 400 μl of 50% acetonitrile, 0.5% trifluoroacetic acid) was applied in two consecutive steps in volumes of 1 μl. Analysis of the retained proteins was performed with a Protein Biological System-IIC (PBSIIC) linear SELDI-TOF-MS instrument (Ciphergen). Mass accuracy was calibrated externally with the all-in-one peptide molecular mass standard (Ciphergen Biosystems, Fremont, CA, USA) for the mass range of 1.6 kDa-20 kDa and with the all-in-one protein molecular mass standard (Ciphergen Biosystems, Fremont, CA, USA) for the mass range of 8-150 kDa.

### Statistical analysis of proteomic data

The SELDI-TOF mass spectra were baseline corrected and normalised on the basis of total ion current using the Biomarker Wizard Program (Ciphergen, Fremont, CA, USA). The same application was used for peak detection and the determination of *p-*values. All univariate analyses were carried out using Ciphergen's ProteinChip Software v3.1.1 (Ciphergen, Fremont, CA, USA) and the Prism 5 software (GraphPad, San Diego, CA, USA). Results are expressed as mean.

### Comparison of proteomics and microarray data

The endometrium samples of the mRNA microarray study which were used in this study revealed that 9 genes were differentially expressed in women with and without endometriosis. The molecular weights of these 9 representative proteins [13] were identified using a search via [21] and are shown in Table 1.

**Table 1:** The representative molecular weights of the proteins identified in the mRNA Microarray study [13]

Protein	Mass in Da
**Osteoglycin (OGN/4969)**	**33,922**
**Interleukin-6 signal transducer (IL6ST/3572)**	isoform 1. 103,537 isoform 2. 37,499
**Cytochrome P450, Family 2, Subfamily J, polypeptide 2 (CYP2J2/1573)**	**57,611**
**Carboxypeptidase E (CPE/1363)**	**53,151**
**Fibronectin 1 (FN1/2335)**	**different isoforms** 1. 262,607 2. 71,943 3. 259,198 4. 222,944 5. 243,316 6. 240,477 7. 268,894 8. 252,793 9. 246,670 10. 239,608 11. 262,388 12. 221,274 13. 249,304 14. 249,384 15. 272,302
**Synuclein, gamma (SNCG/6623)**	**13,331**
**BAI1-associated protein 2 (BAIAP2/10458)**	**different isoforms** 1. 60,868 2. 59,014 3. 56,626 4. 57,359 5. 57,445 6. 57,430
**Protocadherin 17 (PCDH17/27253)**	different isoforms 1. 126,229 2. 96,570

### Results/Discussion

In this study, we showed for the first time that combined analysis of one endometrial sample from women with and without endometriosis for both mRNA (Microarray) and protein fraction (SELDI-TOF MS) is possible after TRIzol extraction. Although we were able to compare these endometrial samples with respect to mRNA protein expression (microarray, [13]) and protein expression (proteomics, presented in this study), no corresponding proteins/genes were found.

In our mRNA microarray study [13] 8 genes were up-regulated and one gene was down-regulated in eutopic endometrium of women with endometriosis compared to controls. Real-time PCR analysis of protocadherin-17 (PCDH17), protein tyrosine phosphatase, receptor type, R (PTPRR) and interleukin-6 signal transducer (IL6ST) expression validated the microarray findings [Table 1; [13]]. In our SELDI-TOF MS study 32 peaks were differentially expressed in endometrium of all women with endometriosis (stages I-IV) compared with all controls during the secretory phase [Table 2]. The proteins of interest detected by SELDI-TOF MS had a lower range (5-32 kDa) than the 9 genes detected by mRNA (representative protein range 33-272 kDa, [13]) with the exception of Synuclein, gamma which has a range of molecular weight of 13 kDa. This observation can be explained by the fact that protein activity often depends on post-translational modifications, which are not predictable from the level of the corresponding transcript [22], and confirm recent data [7] that eutopic endometrial protein expression, analyzed by 2D-differential in gel electrophoresis (DIGE) and mass spectrometry, does not correlate well with published gene array data.

**Table 2:** Mean signal intensities of various proteins and peptides comparing endometrium of women with a normal pelvis versus endometriosis (CM10 and IMAC)

CM10				
**CHCA**				
M/z	pvalue	Mean Disease	Mean Control	Up/down
14653,82	0,034	0,795	1,468	down
16851,18	0,020	0,378	0,660	down
**SPAhigh**				
8776,32	0,020	0,854	0,310	up
8898,40	0,045	4,530	1,935	up
10115,90	0,034	7,442	3,545	up
12186,64	0,026	0,300	0,132	up
12379,95	0,011	2,028	0,420	up
12683,46	0,026	0,988	0,444	up
13464,70	0,045	0,361	0,198	up
14479,03	0,011	0,282	0,116	up
17258,59	0,034	0,370	0,168	up
**SPAlow**				
7662,79	0,045	0,247	0,362	down
8949,82	0,026	1,310	0,720	up
9177,29	0,020	0,391	1,381	down
9941,98	0,026	2,221	1,234	up
10084,76	0,045	1,807	1,096	up
11477,23	0,034	0,087	0,167	down
12323,14	0,011	0,652	0,215	up
12623,72	0,045	0,375	0,219	up
**IMAC**				
**CHCA**				
M/z	pvalue	Mean Disease	Mean Control	Up/down
6299,93	0,011	0,319	0,470	down
9213,51	0,008	0,607	1,578	down
9266,22	0,011	0,933	1,440	down
9766,19	0,034	0,699	0,360	up
11163,87	0,020	0,527	0,245	up
11322,71	0,015	1,017	0,294	up
15446,04	0,004	0,586	0,108	up
**SPAhigh**				
9532,42	0,026	3,217	13,993	down
9767,55	0,045	2,180	4,258	down
**SPAlow**				
7832,06	0,020	0,205	0,369	down
8228,62	0,004	0,389	0,198	up
9190,05	0,015	0,420	1,406	down
9262,94	0,034	0,900	0,502	up

Although it is not clear why different mass peaks were observed in results comparing moderate-severe endometriosis (25 peaks) versus controls as opposed to results comparing minimal-mild endometriosis (23 peaks) versus controls [Table 3 and 4], it is possible that specific peptides or proteins are associated with specific stages of the disease. It is also not clear why the proteomic peaks identified in EM samples in our previous studies [5,6] were not confirmed in the present study. In our first pilot study, [6] SELDI-TOF MS profiling of EM samples showed that the expression of proteins and peptides in the range of 2.8 -12.3 kDa was 3-24 times lower in endometrium of women with endometriosis compared with in women without endometriosis. In our second study [5], the combination of SELDI-TOF MS ProteinChip technology with bioinformatics allowed us to develop a diagnostic test for minimal-mild endometriosis based on a panel of 4 mass peaks (2 up-regulated: 90.675 kDa and 35.956 kDa and 2 down-regulated: 1.9 kDa and 2.5 kDa) with maximal sensitivity (100%) and specificity (100%). We hypothesize that various factors may contribute to this lack of confirmation. Firstly, the protein extraction method was based on TRIzol (Invitrogen Life Technologies, Carlsbad CA, USA) in the current study and on U9 lysis buffer (Ciphergen Biosystems, Fremont, CA) in our previous studies [5,6]. Secondly, endometrial biological changes related to the menstrual cycle [23] may lead to differential protein expression in EM samples obtained on day 23-26 of the cycle (current study), compared to EM samples obtained during secretory phase (day 20-22) or secretory phase (day 16 - 26) in our previous studies [5,6]. Indeed, EM histology on cycle days 23-26 is marked by decreasing secretion, decreasing stromal edema, increasing pseudodecidual reaction, stromal mitoses, and leucocytic infiltration, whereas endometrial secretion on cycle days 18-22 is maximal with a low proportion of stromal mitoses, and absence of pseudodecidual reaction or leucocytic infiltration [23]. Furthermore, endometrial mRNA expression has also been reported to be affected differently during different phases of the cycle [24]. Thirdly, it has to be acknowledged that proteomic techniques like SELDI-TOF MS still require standardization on the level of intra- and interassay variability. Therefore, we plan to repeat this study in a larger sample size including well defined endometrial samples obtained during menstrual, follicular and secretory phase, to validate the reproducibility of SELDI-TOF MS technology in these samples and to identify the protein peaks observed after proteomic analysis, which are expensive and labour intense requiring High-performance liquid chromatography or high-pressure liquid chromatography (HPLC) and matrix assisted laser desorption ionization Time-of-Flight-Mass Spectrometry (MALDI-TOF MS).

**Table 3:** Mean signal intensities of various proteins and peptides comparing endometrium of women with a normal pelvis versus stage I-II endometriosis (CM10 and IMAC)

CM10				
**SPAhigh**				
M/z	pvalue	Mean Disease	Mean Control	Up/down
8898,40	0,036	4,475	1,935	up
10115,90	0,036	8,002	3,545	up
11656,79	0,036	6,899	2,721	up
11861,80	0,008	3,013	1,160	up
12186,64	0,014	0,386	0,132	up
12379,95	0,008	1,580	0,420	up
12847,34	0,023	0,668	0,228	up
13464,70	0,036	0,432	0,198	up
14659,37	0,023	2,197	1,049	up
**SPAlow**				
7662,79	0,036	0,190	0,362	down
8078,54	0,022	0,118	0,339	down
8949,82	0,008	1,326	0,720	up
9941,98	0,008	2,382	1,234	up
10084,76	0,022	1,900	1,096	up
11801,42	0,014	0,875	0,432	up
11835,69	0,008	0,507	0,284	up
12323,14	0,008	0,727	0,215	up
15199,86	0,014	0,010	0,088	down
**IMAC**				
**CHCA**				
M/z	pvalue	Mean Disease	Mean Control	Up/down
11163,87	0,022	0,469	0,245	up
11322,71	0,008	0,836	0,294	up
13815,44	0,014	3,281	1,411	up
15446,04	0,036	0,283	0,108	up
**SPAlow**				
8228,62	0,014	0,429	0,198	up

**Table 4:** Mean signal intensities of various proteins and peptides comparing endometrium of women with a normal pelvis versus stage III-IV endometriosis (CM10 and IMAC)

CM10				
**CHCA**				
M/z	pvalue	Mean Disease	Mean Control	Up/down
9774,53	0,014	0,596	1,098	down
14653,82	0,008	0,445	1,468	down
**SPAhigh**				
14479,03	0,014	0,266	0,116	up
31793,90	0,036	1,596	0,142	up
**SPAlow**				
8172,88	0,014	0,237	0,417	down
8388,16	0,036	0,126	0,362	down
9177,29	0,022	0,145	1,381	down
9399,55	0,036	0,180	0,415	down
9616,30	0,008	0,100	0,307	down
11477,23	0,014	0,058	0,167	down
12623,72	0,036	0,340	0,219	up
12787,28	0,036	0,036	0,132	down
15765,886	0,036	2,103	0,186	up
**IMAC**				
**CHCA**				
M/z	pvalue	Mean Disease	Mean Control	Up/down
6299,9273	0,008113117	0,23461589	0,47046801	down
9213,5098	0,01371083	0,362215739	1,57790751	down
9266,2242	0,022478873	0,695244394	1,44011883	down
9766,1943	0,035763767	0,764862056	0,35977499	up
15446,04	0,008	0,985	0,108	up
**SPAhigh**				
9532,42	0,023	1,056	13,993	down
9767,55	0,036	1,436	4,258	down
13134,421	0,0137	0,23780214	0,53548423	down
**SPAlow**				
7716,86	0,036	0,587	0,253	up
7832,06	0,036	0,204	0,369	down
8228,62	0,022	0,291	0,198	up
9190,0544	0,00811312	0,21041195	1,4064187	down

## Conclusion

TRIzol treatment of secretory phase EM allowed both proteomic (SELDI-TOF MS) and mRNA microarray analysis of the same sample, but comparison of protein and mRNA expression in the same sample was not evident, probably due to post-translational modifications and/or technical aspects.

## Abbreviations

EAM: energy absorbing molecules; EM: endometrium; 2D-differential in gel electrophoresis (DIGE); HPLC: High-performance liquid chromatography or high-pressure liquid chromatography; IMAC: immobilised metal affinity capture; MALDI-TOF/TOF-MS: matrix assisted laser desorption ionization Time-of-Flight-Mass Spectrometry ; PBS IIC: Protein Biological System-IIC; CHCA: alpha-cyano-4-hydroxy cinnamic acid; SPA: sinapinic acid; SELDI-TOF-MS: Surface Enhanced Laser Desorption/Ionisation Time-of-Flight-Mass Spectrometry.

## Competing interests

The authors declare that they have no competing interests.

## Authors' contributions

AF, TD written manuscript, AF, TD, PS, CK, EW, AM, CM, drafting and revising the manuscript and data interpretation, AF, PS, CK, AM, CM, Sample collection, AF, TD, PS, CK, AM design of the experiment, AF, PS, CK experiment, AF, TD, OG, RV, BD, EW data analysis.

All authors read and approved the final manuscript.
